# Auxin transport and conjugation caught together

**DOI:** 10.1093/jxb/erx310

**Published:** 2017-09-30

**Authors:** Kamil Růžička, Jan Hejátko

**Affiliations:** 1 Department of Functional Genomics and Proteomics, CEITEC-Central European Institute of Technology and National Centre for Biomolecular Research, Masaryk University, Kamenice, Brno, Czech Republic; 2 Laboratory of Hormonal Regulations in Plants, Institute of Experimental Botany, Czech Academy of Sciences, Rozvojová, Prague, Czech Republic

**Keywords:** Arabidopsis, endoplasmic reticulum, *GH3*, IAA conjugation, *PIN* expression, polar auxin transport, transcriptional regulation

## Abstract

This article comments on:

**Kong Q, Ma W, Yang H, Ma G, Mantyla JJ, Benning C.** 2017. The Arabidopsis WRINKLED1 transcription factor affects auxin homeostasis in roots. Journal of Experimental Botany **68,** 4627–4634.

**Polar auxin transport together with local synthesis and turnover are crucial for establishing auxin gradients, which determine an array of plant developmental pathways. Transcription factor-mediated control of the genes involved in these processes is steadily receiving increased attention, including efforts to find regulators determining the expression of major auxin efflux carriers of the PIN family. Now,** Kong *et al.* (2017) **have provided evidence of a direct link between auxin metabolism and transport mediated by PINs that is controlled by the transcription factor WRINKLED1.**

In its major form as indole-3-acetic acid (IAA), the plant hormone auxin drives plant growth and development and controls fundamental cellular processes, such as division, expansion and differentiation. Hence, transport of auxin plays a pivotal role in nearly all aspects of plant development, and efflux carriers of the PIN-FORMED (PIN) family have been described as key components exerting this role. Numerous studies have shown that the polar localization of PINs is a critical vectorial feature of auxin flow in Arabidopsis (Zazimalova *et al.*, 2014). Many older studies had shown hormonal and environmental cues to be major regulators of their expression (Vieten *et al.*, 2005; see also review by Krecek *et al.*, 2009). However, the identification of molecular components determining *PIN* expression levels turned out to be very difficult, and the first detailed molecular mechanisms and protein factors acting upstream of these genes have only been uncovered relatively recently. Even less is known about how *PIN*s are co-regulated with other auxin-relevant targets.

## Regulation of *PIN*s

Among the first factors shown to regulate *PIN* expression was the MADS transcription factor XAANTAL2 (XAL2), also known as AGAMOUS-LIKE 14 (AGL14) (Box 1). It was shown that XAL2, which otherwise regulates meristem proliferation and flowering transition, is required for expression of *PIN4* and *PIN1* (Tapia-López *et al.*, 2008; Garay-Arroyo *et al.*, 2013). Meristem defects in the *xal2* mutant resemble those seen in *pin4* and/or *pin1* knockouts or in their higher order mutant combination, and *xal2* mutants also show reduced free IAA levels and polar auxin transport (Friml *et al.*, 2002; Blilou *et al.*, 2005; Garay-Arroyo *et al.*, 2013).

**Box 1. Summary of evidence for the direct regulation of PIN expression by different transcription factors**

Note that the methods indicated are biochemical or heterologous assays besides genetic or complementation analyses outlined in the main text.

**Table:** 

Transcriptional regulator	Promoter target	Method	Notes	Reference
XAL2 (AGL14)	*PIN4*, *PIN1*	ChIP-qPCR		Garay-Arroyo *et al.* (2013)
BRM	*PIN1*, *PIN2*, *PIN3*, *PIN4* and *PIN7*	ChIP-qPCR	Mediates chromatin association with PIN loci	Yang *et al.* (2015)
PPP1	*PIN2*, *PIN1*	Y1H screen, EMSA		Benjamins *et al.* (2016)
CRF2, 3, 7	*PIN7*	Y1H screen, ChIP-qPCR, transient co-expression *in vivo*		Simaskova *et al.* (2015)
ARF7	*PIN3*	Y1H assay, ChIP-qPCR	Also binds *FLP*	Chen *et al.* (2015)
FLP (MYB124), MYB88	*PIN3*, *PIN7*	Y1H assay, ChIP-qPCR, EMSA		Chen *et al.* (2015), Wang *et al.* (2015)
IDD16	*PIN1*	ChIP-qPCR	Also binds *YUC5* and *TAA1* loci for auxin synthesis	Cui *et al.* (2013)
WRI1	*PIN4*, *PIN5*	EMSA	Also binds to *GH3.3* (auxin conjugation)	Kong *et al.* (2017)

Another transcription factor controlling *PIN* expression, PPP1 (PIN2 PROMOTER BINDING PROTEIN 1), is a plant-specific protein of rather unclear function; it has previously been linked with expression of chloroplast-related genes (Lezhneva and Meurer, 2004; Manavski *et al.*, 2015). It was found using systematic deletions of the promoter of *PIN2* and yeast one-hybrid screening (Box 1). The specific *cis* element it binds is essential for stable expression of *PIN2*. In *ppp1* hypomorphs there is reduced expression of *PIN1* and *PIN2* and an altered gravity response which resembles an agravitropic phenotype of *pin2* loss-of-function mutants (Benjamins *et al.*, 2016). The ATPase BRAHMA (BRM) regulates chromatin remodelling that also occurs in the proximity of *PIN1*, *PIN2*, *PIN3*, *PIN4* and *PIN7* regulatory elements. BRM – directly or indirectly – also regulates transcription of *PLETHORA* (*PLT*) genes (Yang *et al.*, 2015). Coordinated action of both PINs and PLTs is required for maintenance of the root stem cell niche (Aida *et al.*, 2004; Blilou *et al.*, 2005). Accordingly, *brm* loss-of-function mutants show pleiotropic defects, including reduced root meristem size, probably caused by defective maintenance of this subset of cells within the meristem (Yang *et al.*, 2015).

*PIN* genes are also targets of hormonal regulatory circuits. Using a promoter deletion strategy and yeast one-hybrid screening, CYTOKININ RESPONSE FACTORS (CRFs) 2, 3 and 7 of the APETALA 2 (AP2, a class ERF VI) family of transcription factors have been demonstrated to regulate expression of *PIN7*. CRFs specifically recognize *PIN CYTOKININ RESPONSE ELEMENT* (*PCRE*) and deletion of this motif from the *PIN7* promoter leads to insensitivity of *PIN7* to cytokinins. In the same line, multiple *crf* loss-of-function mutants display phenotypes similar to those of *pin7* mutants and higher order *pins* (Simaskova *et al.*, 2015).

It has been known for a long time that *PIN* expression can be modulated rapidly by exogenously applied auxins (Vieten *et al.*, 2005). Using the chromatin immunoprecipitation (ChIP) assay technique, both Chen *et al.* (2015) and Wang *et al.* (2015) found that the widely studied AUXIN RESPONSE FACTOR 7 (ARF7) in concert with the MYB transcription factor FOUR LIPS (FLP, MYB124), and partially with FLP paralogue MYB88, directly regulates expression of closely related *PIN3* and *PIN7*. Moreover, FLP is itself a direct target of ARF7. Accordingly, genetic and biochemical approaches, supported by mathematical modelling, revealed that both ARF7 and FLP are required for *PIN3*-mediated lateral root development (Chen *et al.*, 2015; Wang *et al.*, 2015).

Naturally, transcriptional regulators that, besides *PIN*s, also regulate other genes involved in auxin-dependent processes are important. However, only one example of such regulation has been provided to date. INDETERMINATE DOMAIN (IDD) transcription factors belong to the plant-specific family of developmentally important transcriptional regulators (Cui *et al.*, 2013; Long *et al.*, 2015; Yang *et al.*, 2015). *IDD14*, *IDD15* and *IDD16* are, among other processes, required for inflorescence and silique formation and their (ortho)gravitropic responses. It has been reported that IDD16 and possibly IDD14 bind to the promoters of *PIN1* and of genes required for auxin synthesis, namely *YUCCA (YUC) 5* and *TRYPTOPHAN AMINOTRANSFERASE OF ARABIDOPSIS (TAA) 1*. Consequently, *idd* multiple mutants show several auxin-related defects, including altered levels of free IAA and moderately reduced ability to transport auxin (Cui *et al.*, 2013).

## WRINKLED1 (WRI1) regulation

IAA conjugation is a highly important auxin deactivation process and, in contrast to auxin synthesis, our knowledge of this pathway is relatively limited. It is known that most of the total plant IAA pools are present as low molecular weight IAA conjugates with sugars or amino acids (Ludwig-Müller, 2011). Several members of the *GRETCHEN HAGEN 3 (GH3*) gene family encode auxin-inducible acyl amido synthetases required for IAA conjugation with amino acids (Staswick *et al.*, 2005). Free IAA can be released back from some of the IAA conjugates by the action of IAA-amido hydrolases. Although *GH3* genes are classically associated with early auxin transcriptional responses (Hagen and Guilfoyle, 2002), no direct upstream regulator of their expression had been identified until now.

Kong and co-authors have now identified WRINKLED 1 (WRI1) as a possible upstream regulator, coupling both auxin conjugation and transport (Kong *et al.*, 2017). This AP2 transcription factor (a class AP2 ANT) is required for controlling fatty acid and oil synthesis (Cernac and Benning, 2004). The present study reveals that WRI1 binds to the promoter of *GH3.3* in electrophoretic mobility shift assays (EMSAs). Among other *GH3* transcripts, expression of *GH3.3* genes is particularly elevated in the *wri1-1* mutant. This is accompanied by higher content of the IAA-Asp conjugate, while the levels of free IAA levels remain unchanged. Interestingly, the authors also show that WRI1, besides a non-canonical WRI1-binding motif in the *GH3.3* promoter, also binds to promoters of *PIN4* and *PIN5* (but not to *PIN1* and *PIN6* in their experimental setup). Consequently, the expression of several *PIN* genes (*PIN1*, *PIN3*, *PIN5* and *PIN6*) is reduced in the *wri1-1* background. In agreement with these data, sensitivity to exogenously applied auxin and polar auxin transport are also affected in the *wri1-1* mutant (Kong *et al.*, 2017).

Several auxin transport facilitators, including the subclass of so-called short PINs (PIN5, 6 and 8), reside at the endoplasmic reticulum (ER) (Mravec *et al.*, 2009; Barbez *et al.*, 2012). Overexpression of *PIN5* in BY-2 cells leads to increased levels of IAA-Asp and IAA-Glu (products of irreversible conjugation; Östin *et al.*, 1998; Kowalczyk and Sandberg, 2001) at the expense of free IAA. It was proposed that PIN5 might enhance the transport of IAA from the cytoplasm to the ER, which might impede intercellular IAA transport by ER-located auxin degradation (Mravec *et al.*, 2009; Simon *et al.*, 2016). Importantly, Kong *et al.* observed up-regulation of *GH3.3* expression but a drop in *PIN5* expression in *wri1-1* mutants. This suggests a possible link between the activity of GH3.3 and (PIN-mediated) intracellular auxin compartmentalization. In line with this, several IAA-amino acid hydrolases were recently shown to localize to the ER (Sanchez Carranza *et al.*, 2016). The shared transcriptional dependency of both *PIN5* and *PIN6* with *GH3.3* (Kong *et al.*, 2017) leads to speculation as to whether conveying auxin into the ER might enhance the rapidity of specific, irreversible auxin deactivation in this regulatory pathway in a WRI1-dependent manner. Moreover, although the role of fatty acid synthesis has been proposed to interfere with auxin transport, this largely concerned polarity and subcellular trafficking dynamics of PINs (Roudier *et al.*, 2010; Markham *et al.*, 2011). Thus, the link between transcriptional regulation of *PIN* expression and fatty acid synthesis would be an interesting topic for future research.

## Note Added in Proof

While this article was in press, Simonini et al. (2017) revealed, using genome wide approaches, that also ARF3 protein, among others, directly regulates expression of PINs along with the genes required for auxin synthesis.
